# Crossed cerebellar diaschisis in the setting of a convulsive status epilepticus: a rare clinical and radiological entity

**DOI:** 10.1016/j.radcr.2021.06.092

**Published:** 2021-08-01

**Authors:** Rachid Belfkih, Omar Ghomari Khayat, Abdellatif Berkaoui, Hicham Fadel

**Affiliations:** a Abdelmalek Saadi University, Faculty of medicine and pharmacy of Tangier, Tangier, Morocco; b Department of neurology, University Hospital Center of Tangier-Tetouan-Al hoceima, Tangier, Morocco; c Department of neurology at Kortobi Hospital, Tangier, Morocco

**Keywords:** Diaschisis, Status epilepticus, Cerebellum

## Abstract

Crossed cerebellar diaschisis is a rare clinical entity of hemispheric cerebellar depression subsequent to a contralateral cerebral cortical lesion, described to be the result of excessive neuronal excitatory synaptic activity within cortico-cerebellar pathways. This event is generally observed in ischemic stroke cases, and only occasionally, it has been described in epileptic seizure disorders. In this report, we present the case of a patient admitted for status epilepticus with residual motor and visual deficit, with reduced diffusion at DWI. The clinical evolution of her case was distinguished by a full recovery of her deficits along with the disappearance of the MRI abnormalities.

## Introduction

The term diaschisis was first introduced by Von Monakow in 1914 to define the different neurophysiological changes that take place distant from a focal brain injury [1]. Crossed cerebellar diaschisis (CCD) represents depression of local perfusion, metabolism and neuronal activity in the contralateral cerebellar hemisphere in regard to the presumed diseased cerebral cortical area. This event has been typically described in ischemic strokes, and exceptionally, it has been reported in patients with status epilepticus [2]. Herein, we present a clinical case of a patient that was admitted to the emergency room for tonic-clonic seizures, and whose brain DWI MRI revealed reduced diffusion in right temporal, parietal and occipital cortices, the right thalamus and the left cerebellum.

## Case report

The patient is 60 year-old, right-handed women, with a medical history of controlled diabetes mellitus and high blood pressure. She was presented to the emergency room with tonic-clonic seizures that prolonged to 33 minutes, her condition was consistent with convulsive status epilepticus and was therefore transferred to the intensive care unit where she was put on phenobarbital to control her seizures. Once her condition was stabilized, vital parameters such as pulse, respiratory rate, blood pressure and temperature were within standard values. On neurological exam, the patient was conscious, responsive with preserved comprehension and Glasgow coma scale of 15. She presented though a left upper motor neuron syndrome, left central facial palsy and homonymous hemianopsia. There was no tremor or any other involuntary movements, and coordination exam was normal. The rest of the physical exam revealed no abnormality.

**Fig. 1:**
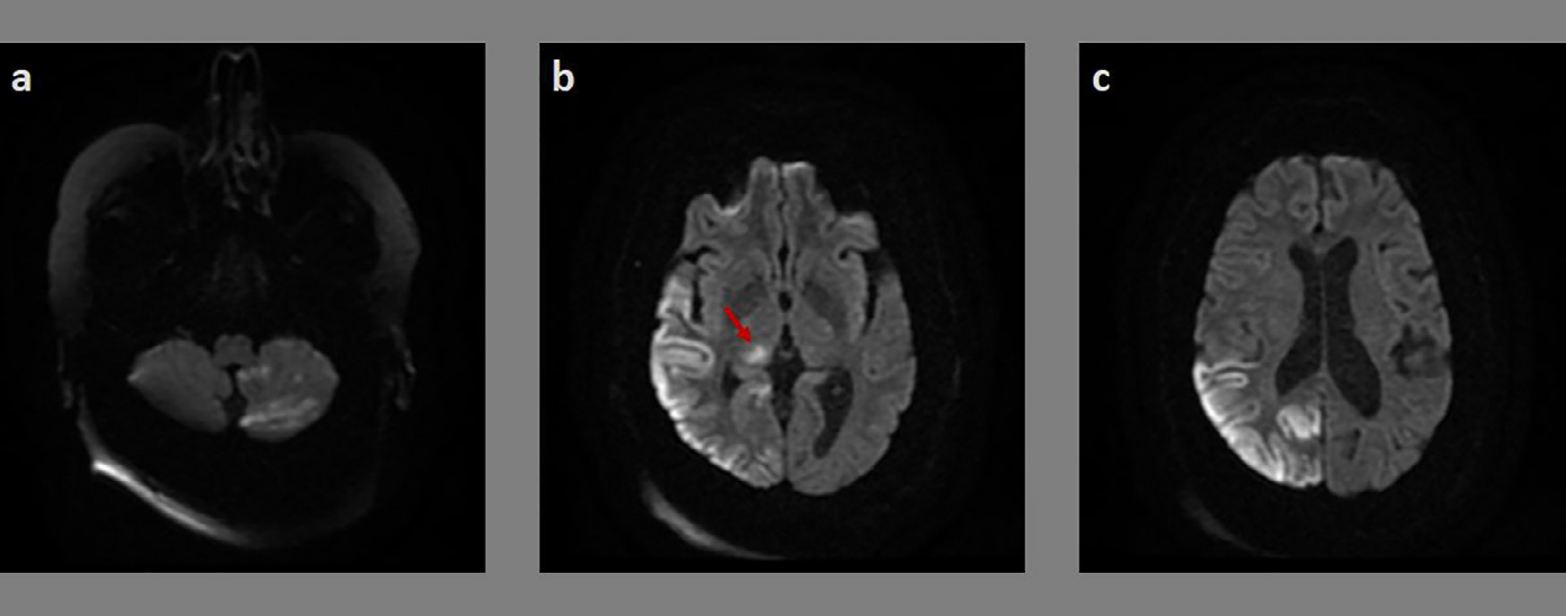
Axial brain DWI MRI showing increased DWI signal in the left cerebellar hemisphere (A), the right temporal lobe and pulvinar thalamic nuclei (red arrow) (B), and the right occipital lobe (C). (Color version of figure is available online.)

The patient has benefited from a brain magnetic resonance imaging (MRI). It showed on DWI spread areas of reduced diffusion signal in right temporal, parietal and occipital cortices, right pulvinar thalamic nuclei and contralateral cerebellar hemisphere (Fig. 1). On fluid attenuated inversion recovery (FLAIR) and T2 sequences, it revealed hyper-signal images in the same before-mentioned areas. The supra-aortic trunks ultrasound showed atheromatous buildups with no significant stenosis. The signal changes revealed on brain MRI images did not answer to a precise vascular territory and were thus correlated to the seizing activity of the convulsive status epilepticus that the patient has endured. Post-ictal EEG continued to show right temporo-parietal and occipital periodic discharges. The overall clinical and radiological presentation was consistent with crossed cerebellar diaschisis in the setting of a convulsive status epilepticus. Thereafter, the patient was discharged and put on antiplatelet therapy with acetylsalicylic acid in prevention of thromboembolic events, and carbamazepine in order to control and prevent further seizures.

**Fig. 2:**
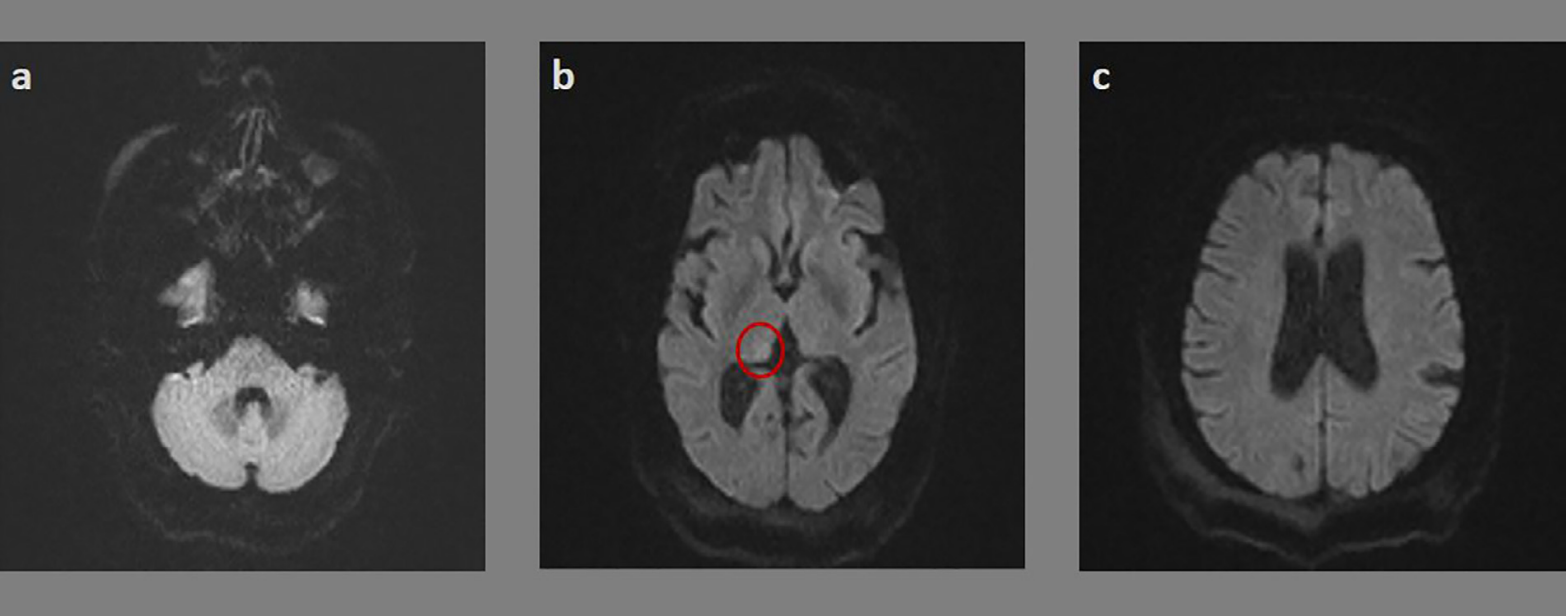
Follow-up brain DWI MRI revealing complete resolution of cerebral cortical and cerebellar signal changes (A, B, C), and reduced hyper-signal in the right pulvinar thalamic nuclei (red circle) (C) compared to the prior images. (Color version of figure is available online.)

At four weeks follow-up, the patient has made, unexpectedly, a good recovery from her neurological deficits. She has benefited as well from a second brain MRI that showed on DWI sequence (Fig. 2) a complete resolution of the previously noted cerebral cortical and cerebellar signal changes, along with reduced hyper-signal in the right pulvinar thalamic nuclei compared to the prior images. Posteriorly, these findings have ruled out an ischemic stroke as a causal etiology of the crossed cerebellar diaschisis in favor of the epileptic event.

## Discussion

Crossed cerebellar diaschisis physiopathology is believed to be a result of excessive transmission input coming from a remote epileptic site to the contralateral cerebellar hemisphere via, predominantly, the cortico-ponto-cerebellar pathways [2]. Whereas, our case is one of the very few that documents a cortico-thalamic-cerebellar circuit. The hyperexcitatory neuronal transmission would be the cause of an impaired balance between cerebellar blood flow and metabolism resulting in tissue hypoxia, anaerobic glycolysis and sodium and potassium pump failure leading to cytotoxic edema [3]. The forenamed disorders would lead to neural depression with its according neurological deficits, and in some cases, neurodegeneration of the ipsilateral thalamus or contralateral cerebellar hemisphere [4].

On DWI and T2 MRI sequences, CCD could be visualized as signal changes in anatomical areas functionally connected to a supratentorial epileptic site. This event could also be detected in perfusion MRI, perfusion CT, fluor-deoxyglucose positron emission tomography (PET), or single photon emission computed tomography (SPECT) [4]. In our case report, diagnosis was made solely on brain MRI input. Since the clinical presentation and radiographic findings were highly suggestive of CCD, resorting to other imaging exams was not required.

Prognostically, patients suffering from seizure-associated CCD usually experience complete clinical recovery and radiological resolution in the following weeks after controlling their seizures [3], as was exactly observed in our case. Rarely, persistent neurological deficits, despite well-conducted medical treatment with complete seizure control, have also been described in medical literature [5]. In another case report similar to ours, where the CCD has involved the cortico-thalamic-cerebellar pathway, improvement of restricted diffusion in the thalamus and cerebellum has been observed. Whereas cerebral abnormalities, similar to what seen in chronic post infarct MRI images, have persisted in the follow-up imaging exams which puts forward the prospect of a cytotoxic damage resulting in neuronal death in status epilepticus associated CCD [2].

We believe that our present case report provides instructive insights on the clinical and radiological presentation of an extremely rare clinical entity along with its clinical evolution, which hopefully would guide physicians in dealing with similar cases, and be of use in large-scale studies that aim to elucidate and set clear guidelines for a better clinical management.

## Patient Consent

Informed written consent was obtained for publication of the findings related to this patient's diagnosis.
